# Few-shot drug synergy prediction via rapid cross-tier adaptation meta-optimization

**DOI:** 10.1093/bib/bbaf683

**Published:** 2025-12-17

**Authors:** Yue-Hua Feng, Ze-Lin Feng, Xiao-Ying Yan, Shao-Wu Zhang, Jian-Yu Shi

**Affiliations:** College of Computer Science, Xi’an Shiyou University, 18 Dianzi'er Road, Yanta District, Xi’an 710065, China; College of Computer Science, Xi’an Shiyou University, 18 Dianzi'er Road, Yanta District, Xi’an 710065, China; College of Computer Science, Xi’an Shiyou University, 18 Dianzi'er Road, Yanta District, Xi’an 710065, China; School of Automation, Northwestern Polytechnical University, 127 Youyi West Road, Beilin District, Xi’an 710072, China; School of Life Sciences, Northwestern Polytechnical University, 127 Youyi West Road, Beilin District, Xi’an 710072, China

**Keywords:** drug synergy prediction, cancer cell lines, few-shot learning, cross-tier meta optimization

## Abstract

Drug combination therapy offers key advantages over monotherapy in personalized oncology by reducing drug resistance and toxicity. However, predicting synergistic effects for rare cell lines remains challenging, as existing methods suffer from poor generalizability in data-scarce scenarios owing to their reliance on large training datasets and inability to effectively transfer knowledge across distinct cellular contexts. Here, we present MetaSynergy, a Rapid Cross-tier Adaptation Meta-Optimization (R-CAMO)-based framework for few-shot drug synergy prediction through cross-domain knowledge transfer and meta-optimized adaptation. We first designed a multimodal feature learning architecture integrating drug molecular graphs with cell line omics profiles, then implemented a stage-wise training strategy based on R-CAMO for few-shot drug synergy prediction: (i) cross-domain pretraining establishes meta-initialized representations by transferring knowledge from data-rich cell lines to scarce target domains, enhancing feature representation capability in data-scarce scenarios. (ii) Cross-tier meta-optimization enables rapid adaptation to data-scarce scenarios: the inner-tier refines task-specific parameters of the prediction network on the target domain, while the outer-tier meta-learns task-shared, generalizable parameters by minimizing the cross-cell line prediction loss. (iii) Fine-tuning further refines task-specific parameters, improving generalizability to novel drug combinations within the same cellular context. Experimental results demonstrate that MetaSynergy achieves excellent performance in few-shot, zero-shot and low-similarity tasks, surpassing most baseline methods and highlighting its robustness and generalizability. Ablation studies confirmed the pivotal role of R-CAMO strategy in data-scarce cell lines. Furthermore, MetaSynergy successfully identified novel synergistic drug combinations in several understudied malignancies, underscoring its potential in precision oncology.

## Introduction

Drug combination therapy is a fundamental strategy for treating malignancies and multifactorial diseases [1]. Owing to the limitations of monotherapy regimens in comprehensively targeting heterogeneous cancer cell mutations and adaptive resistance mechanisms, multi-drug regimens not only mitigate adverse effects and overcome drug resistance but also enhance therapeutic efficacy through synergistic interactions. High-throughput drug combination screening [2], a systematic approach for identifying synergistic drug pairs, facilitates the simultaneous evaluation of combinatorial drug responses in various cancer cells. However, as the number of candidate drugs increases, the exponential growth in potential drug combinations makes experimental testing exceedingly time-consuming and cost-prohibitive, ultimately rendering comprehensive screening of all possible combinations infeasible. Consequently, the development of efficient and accurate computational methods for predicting drug synergy is crucial for expediting the identification of novel therapeutic strategies.

Computational prediction of drug synergy has advanced with the development of large-scale pharmacogenomic resources such as DrugCombDB [3], which provides standardized data on drug properties and cell line omics profiles [4]. Early approaches relied on traditional machine learning methods (e.g. RF, SVM, XGBoost) using handcrafted features [5, 6]; however, these models lacked the capacity to capture complex drug-cell line interactions.

Recent efforts have shifted toward deep learning, which automatically learns hierarchical representations from raw data. One class of models uses multi-layer perceptron (MLP) to integrate chemical descriptors and transcriptomic profiles, exemplified by DeepSynergy [7] and MatchMaker [8]. Others extend these with tensor factorization [9] or dimensionality reduction [10] to enhance their expressiveness. Another key direction in drug synergy prediction involves geometric deep learning, particularly graph neural networks (GNNs), which model drugs as molecular graphs where atoms and bonds are represented as nodes and edges, respectively. These methods capture structural dependencies using graph convolutional networks (GCNs) for neighborhood aggregation [11]. Extensions have enhanced biological relevance by incorporating protein–protein interaction (PPI) networks [12], multimodal integration of cellular and molecular features [13], and temporal dynamics using GCN–long short-term memory architectures [14]. Recent advances in heterogeneous graph modeling [15, 16] and ensemble learning [17, 18] have demonstrated strong performance in data-rich biological network analysis. However, these models require extensive labeled datasets, which are scarce for rare cancer cell lines (e.g. bone and prostate cancer). This scarcity stems from technical and biological limitations in cell line cultivation and profiling, which restrict generalizability.

Transfer learning partially mitigates data scarcity by leveraging knowledge from data-rich domains, which involves transferring pretrained parameters or learned representations to target tasks. For example, Kim *et al.* [19] fine-tuned models from common to rare cancers, while Li *et al.* [20, 21] employed domain adaptation and cross-species transfer via selective parameter freezing. Nevertheless, transfer learning depends on sufficient labeled data in target domains and presupposes domain similarity—assumptions that often do not hold in the context of heterogeneous cancer types.

Few-shot learning (FSL) has recently emerged as a promising alternative for addressing these limitations. By leveraging meta-learning, FSL enables rapid adaptation across tasks with limited labeled data through episodic training on diverse task distributions. Model-Agnostic Meta-Learning (MAML) [22] and its derivative algorithms [23–26] have demonstrated exceptional performance in FSL tasks. For instance, Guo *et al.* [27] applied MAML to few-shot molecular property prediction, enabling rapid adaptation to new tasks with limited samples by transferring knowledge across related property prediction tasks. However, MAML suffers from gradient instability in high-dimensional space. Zhang *et al.* [28] proposed a deep Bayesian variational framework that learns prior distributions over task embeddings to generate cell line–specific model parameters. Although this approach improves adaptability across cell lines, its architectural complexity introduces significant computational overheads during training and inference. In parallel, the transformer-based large language model CancerGPT [29] leverages large-scale pretrained biomedical text to enable drug synergy prediction in data-scarce scenarios. However, these methods either face optimization instability or require intensive computational resources.

To address the key challenges of drug synergy prediction in data-scarce scenarios, we propose MetaSynergy, a R-CAMO-based few-shot drug synergy prediction model. Unlike previous models relying on shallow or unimodal representations, MetaSynergy adopts a multimodal deep-representation learning architecture that combines a GCNs to hierarchically aggregate structural features from drug molecular graphs and a convolutional neural network (CNN) to extract transcriptomic features from gene expression profiles of cancer cell lines, fusing their heterogeneous representations into a unified drug-cell line embedding for synergy prediction. Unlike conventional meta-learning methods utilizing uniform parameter adaptation, MetaSynergy introduces a principled stage-wise training strategy for data-scarce scenarios, enabling selective parameter optimization through three progressive stages: (i) cross-domain pretraining that transfers knowledge from data-rich to scarce cell lines to establish meta-initialized representations; (ii) cross-tier meta-optimization with inner-tier task-specific adaptation and outer-tier generalizable parameter learning; and (iii) target-aware fine-tuning to enhance generalizability to novel drug combinations. Comprehensive experimental results show that MetaSynergy consistently outperforms most baseline methods in few-shot (with 5/10/30 samples), zero-shot and low-similarity tasks, demonstrating superior robustness and generalizability. Ablation studies further validated the pivotal role of R-CAMO strategy in enhancing prediction accuracy in data-scarce scenarios. Additional analysis revealed a significant positive correlation between inter-cell-line heterogeneity and model prediction errors. Notably, MetaSynergy successfully identified novel synergistic drug combinations in several understudied malignancies, demonstrating its potential for precision oncology applications in rare cancers.

## Materials and methods

### Datasets

Drug synergy data were collected from SYNERGxDB [30], a comprehensive pharmacogenomic repository consolidating large-scale drug combination screening datasets. Following the preprocessing protocol of Zhang *et al.* [28], we curated a final dataset comprising 427 710 drug combination-cell line pairs involving 1928 drugs and 106 cancer cell lines. As summarized in Table 1, the filtered dataset captures the number of unique drugs, unique drug combinations, cell lines, drug combination-cell line pairs, and the dimensions of the dose–response matrix per drug pair across individual screening studies. Molecular structures were downloaded from PubChem in SDF format and transformed into molecular graphs using RDKit. Gene expression profiles for all cancer cell lines were sourced from the Cancer Cell Line Encyclopaedia (CCLE) [31]. Further dataset details are provided in Supplementary Appendix A.1.

**Table 1:** Statistics of the processed drug synergy dataset.

Source	No. of drugs	No. of cell lines	No. of drug pairs	No. of samples	Dose of drug pairs
YALE-PDAC	39	3	780	2228	1 × 5
MIT-MELANOMA	97	24	4656	112,099	2 × 2
YALE-TNBC	122	6	720	4508	1 × 5
MERCK	38	37	583	22,253	4 × 4
DECREASE	33	10	36	187	8 × 8
VISACE	2	29	1	29	10 × 6
STANFORD	1816	8	1816	1816	1 × 1
NCI-ALMANAC	101	58	5151	287,519	3 × 3 or 5 × 3
Total (unique, validation)	1928	106	13,246	427,710	–

### Problem formulation

We define two disjoint sets of cell lines: a base set ${\boldsymbol{C}}_{\boldsymbol{b}}$ with abundant drug synergy samples ${\boldsymbol{D}}_{\boldsymbol{b}}=\left\{\left(\boldsymbol{z},\boldsymbol{y}\right)|\boldsymbol{z}\boldsymbol{\in }{\boldsymbol{Z}}_{\boldsymbol{b}},\boldsymbol{y}\in \mathbb{R}\right\}$ and a new cell lines set ${\boldsymbol{C}}_{\boldsymbol{n}}$ with limited samples ${\boldsymbol{D}}_{\boldsymbol{n}}=\left\{\left(\boldsymbol{z},\boldsymbol{y}\right)|\boldsymbol{z}\boldsymbol{\in }{\boldsymbol{Z}}_{\boldsymbol{n}},\boldsymbol{y}\in \mathbb{R}\right\}$, where ${\boldsymbol{Z}}_{\boldsymbol{b}}$ and ${\boldsymbol{Z}}_{\boldsymbol{n}}$ denote the feature space of drug synergy samples in cell lines ${\boldsymbol{C}}_{\boldsymbol{b}}$ and ${\boldsymbol{C}}_{\boldsymbol{n}}$, respectively; $\boldsymbol{z}$ denotes the sample feature vector generated by deep learning module, $\boldsymbol{y}$ denotes the drug synergy score. Our goal is to design an FSL model that can leverage the limited drug synergy samples from new cell lines to accurately predict synergy scores of unseen drug combinations.

Formally, given a set of base cell lines ${\boldsymbol{C}}_{\boldsymbol{b}}$ and their drug synergy samples ${\boldsymbol{D}}_{\boldsymbol{b}}$, and a set of new cell lines ${\boldsymbol{C}}_{\boldsymbol{n}}$ and their drug synergy samples ${\boldsymbol{D}}_{\boldsymbol{n}}=\left\{\left({\boldsymbol{z}}_{\boldsymbol{n}\boldsymbol{j}},{\boldsymbol{y}}_{\boldsymbol{n}\boldsymbol{j}}\right)|{\boldsymbol{z}}_{\boldsymbol{n}\boldsymbol{j}}\boldsymbol{\in}{\boldsymbol{Z}}_{\boldsymbol{n}\boldsymbol{j}},{\boldsymbol{y}}_{\boldsymbol{n}\boldsymbol{j}}\in \mathbb{R}\right\}$, $\boldsymbol{j}\boldsymbol{\in}\left\{\mathbf{1},\mathbf{2},\boldsymbol{\cdots},\left|{\boldsymbol{C}}_{\boldsymbol{n}}\right|\right\}$, the goal is to learn a meta-function $\boldsymbol{F}:{\boldsymbol{D}}_{\boldsymbol{j}}\boldsymbol{\to}{\boldsymbol{f}}_{\boldsymbol{j}}$ with ${\boldsymbol{D}}_{\boldsymbol{b}}$. Subsequently, the few samples ${\boldsymbol{D}}_{\boldsymbol{nj}}$ are used to rapidly learn a regression function ${\boldsymbol{f}}_{\boldsymbol{nj}}:{\boldsymbol{Z}}_{\boldsymbol{nj}}\to \mathbb{R}$ to predict the synergy scores of other drug combinations for each new cell line ${\boldsymbol{c}}_{\boldsymbol{j}}\boldsymbol{\in}{\boldsymbol{C}}_{\boldsymbol{n}}$.

### MetaSynergy model

**Figure 1:**
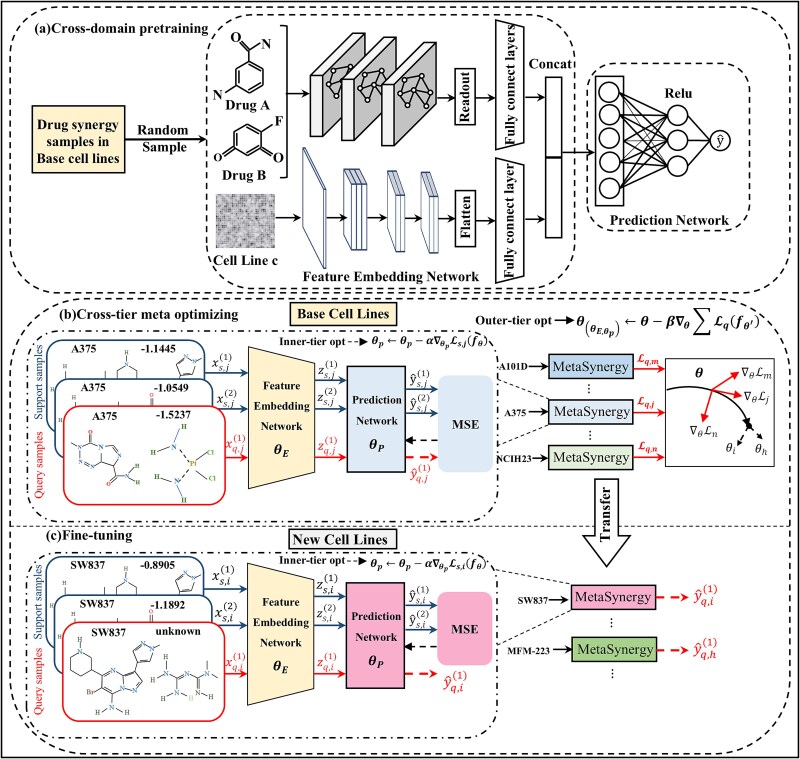
Overview of MetaSynergy. MetaSynergy consists of two modules: the feature embedding network that learns discriminative representations from drug-cell line data, and the prediction network that maps these representations to quantitative synergy scores. The model is trained through a stage-wise training strategy: (a) cross-domain pretraining. The feature embedding network is pretrained using drug synergy data from base cell lines, these optimized parameters initialize the subsequent cross-tier optimization, enhancing the model’s feature representation capability for data-scarce scenarios. (b) Cross-tier meta optimization: for each few-shot drug synergy prediction task on a given cell line, the inner-tier loop optimization generates task-specific prediction network parameters based on the support set. The outer-tier loop optimization then updates a shared meta-initialization by minimizing the query losses across multiple cell lines, thereby enhancing the model’s generalization ability to unseen tasks. (c) Fine-tuning: it leverages few-shot target cell line samples to further refine task-specific parameters, allowing rapid adaption to prediction tasks for these cell lines.

MetaSynergy is an FSL framework based on R-CAMO, designed for drug synergy prediction in data-scarce cell lines. The architecture integrates a multimodal feature embedding network that extracts biologically discriminative representations from cell line omics profiles and drug combination features, and a meta-optimized prediction network that maps these integrated representations to quantitative synergy scores. To enable rapid adaptation with limited data, MetaSynergy adopts a stage-wise training strategy to implement R-CAMO: (i) cross-domain pretraining (Fig. 1a) optimizes the feature embedding parameters ${\boldsymbol{\theta}}_{\boldsymbol{E}}$ through large-scale training on base cell lines; (ii) cross-tier meta optimization (Fig. 1b) with inner-tier task-specific adaptation of prediction parameters ${\boldsymbol{\theta}}_{\boldsymbol{p}}$ via gradient descent and outer-tier learns task-shared, generalizable parameters by minimizing the cross-cell line prediction loss; (iii) Fine-tuning (Fig. 1c) transfers meta-knowledge to new cell lines by adjusting ${\boldsymbol{\theta}}_{\boldsymbol{p}}$ with few samples.

#### Model architecture

##### Feature embedding network

The feature embedding network employs a three-layer GCN to hierarchically aggregate neighborhood information, extracting multi-scale structural and attribute features from drug molecular graphs. Each drug molecule is modeled as an undirected graph ${\boldsymbol{G}}_{\boldsymbol{d}}=\left({\boldsymbol{V}}_{\boldsymbol{d}},{\boldsymbol{E}}_{\boldsymbol{d}}\right)$, where nodes ${\boldsymbol{V}}_{\boldsymbol{d}}=\left\{{\boldsymbol{v}}_{\mathbf{1}},\boldsymbol{\cdots},{\boldsymbol{v}}_{\boldsymbol{u}}\right\}$ represents the atoms and edges ${\boldsymbol{E}}_{\boldsymbol{d}}\boldsymbol{\subseteq}{\boldsymbol{V}}_{\boldsymbol{d}}\times{\boldsymbol{V}}_{\boldsymbol{d}}$ represents the chemical bonds between atoms. Each node ${\boldsymbol{v}}_{\boldsymbol{u}}$ is initially represented by a 78-dimensional feature vector ${\boldsymbol{h}}_{\boldsymbol{u}}^{\left(\mathbf{0}\right)}$, encoding atom symbol, adjacent atom count, implicit valency, hydrogen bond count, and aromaticity. At each GCN layer, node features are updated through neighborhood aggregation, and the computation is given by the following formula:


(1) \begin{equation*} {\boldsymbol{h}}_{\boldsymbol{u}}^{\left(\boldsymbol{l}\right)}=\boldsymbol{ReLU}\left({\boldsymbol{W}}^{\left(\boldsymbol{l}\right)}\sum_{\boldsymbol{v}\mathbf{\in}\boldsymbol{N}\left(\boldsymbol{u}\right)\cup \left\{\boldsymbol{u}\right\}}\frac{{\boldsymbol{e}}_{\boldsymbol{v},\boldsymbol{u}}}{\sqrt{{\hat{\boldsymbol{D}}}_{\boldsymbol{v}}{\hat{\boldsymbol{D}}}_{\boldsymbol{u}}}}{\boldsymbol{h}}_{\boldsymbol{v}}^{\left(\boldsymbol{l}-\mathbf{1}\right)}\right) \end{equation*}

where $\boldsymbol{N}\left(\boldsymbol{u}\right)$ denotes the set of neighbors of node $\boldsymbol{u}$, ${\boldsymbol{e}}_{\boldsymbol{v},\boldsymbol{u}}$ represents the edge weight from node $\boldsymbol{v}$ to $u$, ${\hat{\boldsymbol{D}}}_{\boldsymbol{u}}=\mathbf{1}+\sum_{\boldsymbol{v}\boldsymbol{\in}\boldsymbol{N}\left(\boldsymbol{u}\right)}{\boldsymbol{e}}_{\boldsymbol{v},\boldsymbol{u}}$ is the weighted degree of node $u$, and ${\boldsymbol{W}}^{\left(\boldsymbol{l}\right)}$ is the transformation matrix used to update the node embedding representations.

The final graph-level embedding ${\boldsymbol{h}}_{\boldsymbol{d}}$ is obtained by applying a readout function that performs max pooling over all node embeddings in the last GCN layer:


(2) \begin{equation*} {\displaystyle \begin{array}{c}{\boldsymbol{h}}_{\boldsymbol{d}}=\boldsymbol{\operatorname{Re}}\mathbf{a}\boldsymbol{dout}\left({\boldsymbol{h}}_{\boldsymbol{u}}^{\left(\boldsymbol{l}\right)}|{\boldsymbol{v}}_{\boldsymbol{u}}\mathbf{\in}{\boldsymbol{V}}_{\boldsymbol{d}}\right)\end{array}} \end{equation*}

where the readout function selects the maximum value across nodes for each feature dimension to yield a comprehensive molecular representation.

To capture biologically relevant patterns from cell line transcriptomes, we structure each profile as a 30 × 30 matrix representing 900 drug response-associated marker genes, arranged based on prior biological knowledge. This spatial organization allows the CNN to exploit local receptive fields for detecting localized gene expression motifs, while parameter sharing across spatial dimensions reduces model complexity. The multi-layer CNN extracts hierarchical features from this input. The output of the $\boldsymbol{L}$-th convolutional layer is computed as:


(3) \begin{equation*} {\displaystyle \begin{array}{c}{\boldsymbol{h}}_{\boldsymbol{c}}^{\left(\boldsymbol{L}\right)}=\mathbf{P}\boldsymbol{ool}\left(\boldsymbol{ReLU}\left(\boldsymbol{BN}\left({\boldsymbol{W}}_{\boldsymbol{C}}^{\left(\boldsymbol{L}\right)}\ast{\boldsymbol{h}}_{\boldsymbol{c}}^{\left(\boldsymbol{L}-\mathbf{1}\right)}+{\boldsymbol{b}}_{\boldsymbol{C}}^{\left(\boldsymbol{L}\right)}\right)\right)\right)\end{array}} \end{equation*}

where ${\boldsymbol{W}}_{\boldsymbol{C}}^{\left(\boldsymbol{L}\right)}$ and ${\boldsymbol{b}}_{\boldsymbol{C}}^{\left(\boldsymbol{L}\right)}$ are the convolutional weights and bias, respectively; $\boldsymbol{BN}\left(\cdot \right)$ represents the batch normalization operation, $\boldsymbol{Pool}\left(\cdot \right)$ indicates the max pooling operation.

##### Feature integration layer

This layer integrates embeddings of a drug pair $\left({\boldsymbol{d}}_{\boldsymbol{A}},{\boldsymbol{d}}_{\boldsymbol{B}}\right)$ and a cell line $\boldsymbol{c}$ from the feature embedding network. The combined embedding $\boldsymbol{z}$ is formed by concatenating transformed features:


(4) \begin{equation*} {\displaystyle \begin{array}{c}\boldsymbol{z}=\boldsymbol{Concat}\left({\boldsymbol{f}}_{\boldsymbol{d}}\left({\boldsymbol{h}}_{\boldsymbol{d}\boldsymbol{A}}\right),{\boldsymbol{f}}_{\boldsymbol{d}}\left({\boldsymbol{h}}_{\boldsymbol{d}\boldsymbol{B}}\right),{\boldsymbol{f}}_{\boldsymbol{c}}\left({\boldsymbol{h}}_{\boldsymbol{c}}\right)\right)\end{array}} \end{equation*}

where ${\boldsymbol{h}}_{\boldsymbol{dA}}$ and ${\boldsymbol{h}}_{\boldsymbol{dB}}$ are drug embeddings from the multi-layer GCN, and ${\boldsymbol{h}}_{\boldsymbol{c}}$ is the cell line $\boldsymbol{c}$ embedding from CNN. The transformation functions ${\boldsymbol{f}}_{\boldsymbol{d}}\left(\cdot \right)$ and ${\boldsymbol{f}}_{\boldsymbol{c}}\left(\cdot \right)$ are fully connected (FC) layers defines as:


(5) \begin{equation*} {\displaystyle \begin{array}{c}{\boldsymbol{f}}_{\boldsymbol{d}}\left({\boldsymbol{h}}_{\boldsymbol{d}}\right)={\boldsymbol{FC}}_{\mathbf{2}}\left(\boldsymbol{ReLU}\left(\boldsymbol{Dropout}\left({\boldsymbol{FC}}_{\mathbf{1}}\left({\boldsymbol{h}}_{\boldsymbol{d}}\right)\right)\right)\right)\end{array}} \end{equation*}

(6) \begin{equation*} {\displaystyle \begin{array}{c}{\boldsymbol{f}}_{\boldsymbol{c}}\left(\cdot \right)=\boldsymbol{FC}\left({\boldsymbol{h}}_{\mathbf{c}}\right)\end{array}} \end{equation*}

$\boldsymbol{Dropout}\left(\cdot \right)$ is applied for regularization, and $\boldsymbol{Concat}\left(\cdot \right)$ denotes the feature concatenation operation.

Prediction Network. The prediction module consists of two FC layers that map the integrated embedding $\boldsymbol{z}$ to the prediction drug synergy score $\hat{\boldsymbol{y}}$:


(7) \begin{equation*} {\displaystyle \begin{array}{c}\hat{\boldsymbol{y}}=\left(\boldsymbol{ReLU}\left(\boldsymbol{z}\ast{\boldsymbol{W}}_{\mathbf{1}}+{\boldsymbol{b}}_{\mathbf{1}}\right)\right){\boldsymbol{W}}_{\mathbf{2}}+{\boldsymbol{b}}_{\mathbf{2}}\end{array}} \end{equation*}

where ${\boldsymbol{W}}_{\mathbf{1}}$, ${\boldsymbol{W}}_{\mathbf{2}}$, ${\boldsymbol{b}}_{\mathbf{1}}$, and ${\boldsymbol{b}}_{\mathbf{2}}$ are learnable weights and biases.

#### Stage-wise training strategy

To enable few-shot drug synergy prediction, we developed a three-stage progressive learning framework to implement R-CAMO: (i) the pretraining stage constructs meta-initialized representations through knowledge transfer from data-rich to data-scarce cellular contexts, thereby enhancing feature representation capabilities. (ii) The cross-tier meta optimization stage applies inner-tier and outer-tier collaborative optimization to the parameters of both the feature embedding network and the prediction network at the task-level with drug synergy samples from base cell lines. (iii) Fine-tuning leverages few-shot target cell line samples to further refine task-specific parameters, improving the generalizability to novel drug combinations within the same cellular context.

##### Cross-domain pretraining stage

The feature embedding network ${\boldsymbol{\theta}}_{\boldsymbol{E}}$ is pretrained on drug synergy samples ${\boldsymbol{D}}_{\boldsymbol{b}}$ from base cell lines to learn generalizable feature representations. During training, drug synergy samples are randomly sampled from the base cell lines, and the model parameters are optimized by minimizing the mean squared error (MSE) between the predicted and true synergy scores. Specifically, given $\boldsymbol{m}$ drug synergy samples $\left({\boldsymbol{X}}_{\boldsymbol{i}},{\boldsymbol{y}}_{\boldsymbol{i}}\right)$, the optimization objective for the pretraining task is defined as follows:


(8) \begin{equation*} {\displaystyle \boldsymbol{\min}\frac{\mathbf{1}}{\mathbf{2}}\sum\limits_{\boldsymbol{i}=\mathbf{1}}^{\boldsymbol{m}}{\left(\boldsymbol{f}\left({\boldsymbol{\theta}}_{\boldsymbol{E}}\left({\boldsymbol{X}}_{\boldsymbol{i}}\right)\right)-{\boldsymbol{y}}_{\boldsymbol{i}}\right)}^{\mathbf{2}}} \end{equation*}

where $f\left({\boldsymbol{\theta}}_{\boldsymbol{E}}\left({X}_i\right)\right)$ denotes the predicted drug synergy score.

##### Cross-tier meta optimization stage

In this stage, the pretrained feature embedding network parameters ${\boldsymbol{\theta}}_{\boldsymbol{E}}$ serve as meta-initialization for MetaSynergy. We construct numerous few-shot drug synergy prediction tasks using the drug synergy samples ${\boldsymbol{D}}_{\boldsymbol{b}}$ from the base cell line ${\boldsymbol{C}}_{\boldsymbol{b}}$, and employ an inner-tier and outer-tier collaborative optimization to enable rapid adapt across cell lines. Specifically, an episode-based iterative training procedure is adopted. In each episode, $\boldsymbol{N}$ cell lines are randomly sampled from ${\boldsymbol{C}}_{\boldsymbol{b}}$. For each cell line, $\boldsymbol{K}$ drug synergy samples are randomly selected as the support set and $\boldsymbol{Q}$ drug synergy samples as the query set to form an FSL task. The support set is defined as ${\boldsymbol{D}}_{\boldsymbol{s},\boldsymbol{j}}=\{({\boldsymbol{x}}_{\boldsymbol{s},\boldsymbol{j}}^{(\boldsymbol{i})},{\boldsymbol{y}}_{\boldsymbol{s},\boldsymbol{j}}^{(\boldsymbol{i})})|\boldsymbol{i}=\mathbf{1},\boldsymbol{\cdots},\boldsymbol{K};\boldsymbol{j}=\mathbf{1},\boldsymbol{\cdots},\boldsymbol{N}\}$, and the query set as ${\boldsymbol{D}}_{\boldsymbol{q},\boldsymbol{j}}=\{({\boldsymbol{x}}_{\boldsymbol{q},\boldsymbol{j}}^{(\boldsymbol{i})},{\boldsymbol{y}}_{\boldsymbol{q},\boldsymbol{j}}^{(\boldsymbol{i})})|\boldsymbol{i}=\boldsymbol{K}+\mathbf{1},\boldsymbol{\cdots},\boldsymbol{K}+\boldsymbol{Q};\boldsymbol{j}=\mathbf{1},\boldsymbol{\cdots},\boldsymbol{N}\}$. MetaSynergy performs task-specific adaptation for each cell line via inner-tier optimization, while the outer-tier optimization updates initialization parameters to ensure generalizability across all cell lines. Concretely, for each cell line ${\boldsymbol{c}}_{\boldsymbol{j}}$, the drug synergy scores ${\mathbf{y}}_{\mathbf{s},\mathbf{j}}^{\left(\mathbf{i}\right)}$ for the support set samples ${\overset{\sim }{\boldsymbol{D}}}_{\boldsymbol{s},\boldsymbol{j}}=\left\{\left({\boldsymbol{x}}_{\boldsymbol{s},\boldsymbol{j}}^{\left(\boldsymbol{i}\right)},{\boldsymbol{y}}_{\boldsymbol{s},\boldsymbol{j}}^{\left(\boldsymbol{i}\right)}\right)|{\boldsymbol{x}}_{\boldsymbol{s},\boldsymbol{j}}^{\left(\boldsymbol{i}\right)}\boldsymbol{\epsilon} {\boldsymbol{X}}_{\boldsymbol{j}},{\boldsymbol{y}}_{\boldsymbol{s},\boldsymbol{j}}^{\left(\boldsymbol{i}\right)}\boldsymbol{\epsilon} \mathbb{R}\right\}\left(\boldsymbol{i}=\mathbf{1},\boldsymbol{\cdots},\boldsymbol{K}\right)$ are known, while the drug synergy scores ${\mathbf{y}}_{\mathbf{q},\mathbf{j}}^{\left(\mathbf{i}\right)}$ for the query set samples ${\overset{\sim }{\boldsymbol{D}}}_{\boldsymbol{q},\boldsymbol{j}}=\left\{\left({\boldsymbol{x}}_{\boldsymbol{q},\boldsymbol{j}}^{\left(\boldsymbol{i}\right)},{\boldsymbol{y}}_{\boldsymbol{q},\boldsymbol{j}}^{\left(\boldsymbol{i}\right)}\right)|{\boldsymbol{x}}_{\boldsymbol{q},\boldsymbol{j}}^{\left(\boldsymbol{i}\right)}\boldsymbol{\epsilon} {\boldsymbol{X}}_{\boldsymbol{j}},{\boldsymbol{y}}_{\boldsymbol{q},\boldsymbol{j}}^{\left(\boldsymbol{i}\right)}\boldsymbol{\epsilon} \mathbb{R}\right\}\left(\boldsymbol{i}=\boldsymbol{K}+\mathbf{1},\boldsymbol{\cdots},\boldsymbol{K}+\boldsymbol{Q}\right)$ are unknown. The support set samples are first fed into the model parameterized by $\boldsymbol{\theta} =\left({\boldsymbol{\theta}}_{\boldsymbol{E}},{\boldsymbol{\theta}}_{\boldsymbol{p}}\right)$. The feature embedding network ${\boldsymbol{\theta}}_{\boldsymbol{E}}:{\boldsymbol{X}}_{\boldsymbol{j}}\boldsymbol{\to}{\boldsymbol{Z}}_{\boldsymbol{j}}$ maps the support set samples ${\mathbf{x}}_{\mathbf{s},\mathbf{j}}^{\left(\mathbf{i}\right)}=\left\{{\left({\mathbf{d}}_{\mathbf{A}},{\mathbf{d}}_{\mathbf{B}}\right)}^{\left(\mathbf{i}\right)},{\mathbf{c}}_{\mathbf{j}}\right\}\left(\mathbf{i}=\mathbf{1},\mathbf{\cdots},\mathbf{K}\right)$(where $\left({\boldsymbol{d}}_{\boldsymbol{A}},{\boldsymbol{d}}_{\boldsymbol{B}}\right)$ represents the drug pair) and query set samples ${\mathbf{x}}_{\mathbf{q},\mathbf{j}}^{\left(\mathbf{i}\right)}=\left\{{\left({\mathbf{d}}_{\mathbf{A}},{\mathbf{d}}_{\mathbf{B}}\right)}^{\left(\mathbf{i}\right)},{\mathbf{c}}_{\mathbf{j}}\right\}(\mathbf{i}=\mathbf{K}+\mathbf{1},\mathbf{\cdots},\mathbf{K}+\mathbf{Q})$ into the feature embedding space, producing corresponding feature embeddings ${\boldsymbol{z}}_{\boldsymbol{s},\boldsymbol{j}}^{\left(\boldsymbol{i}\right)}$ and ${\boldsymbol{z}}_{\boldsymbol{q},\boldsymbol{j}}^{\left(\boldsymbol{i}\right)}$. During the inner-tier optimization, the prediction network ${\boldsymbol{\theta}}_{\boldsymbol{p}}:{\boldsymbol{Z}}_{\boldsymbol{j}}\to \mathbb{R}$ computes the loss function ${\mathbf{\mathcal{L}}}_{\boldsymbol{s},\boldsymbol{j}}\left({\boldsymbol{f}}_{\boldsymbol{\theta}}\right)$ based on the support set samples ${\overset{\sim }{\boldsymbol{D}}}_{\boldsymbol{s},\boldsymbol{j}}$. The prediction network parameters ${\boldsymbol{\theta}}_{\boldsymbol{p}}$ are updated via gradient descent to obtain the adapted parameters ${\boldsymbol{\theta}}_{\boldsymbol{j}}^{\prime }$ specific to the drug synergy prediction task for cell line ${\boldsymbol{c}}_{\boldsymbol{j}}$. The update rule is as follows:


(9) \begin{equation*} {\displaystyle \begin{array}{c}{\boldsymbol{\theta}}_{\boldsymbol{j}}^{\prime }=\left({\boldsymbol{\theta}}_{\boldsymbol{E}},{\boldsymbol{\theta}}_{\boldsymbol{p}}-\boldsymbol{\alpha} {\boldsymbol{\nabla}}_{{\boldsymbol{\theta}}_{\boldsymbol{p}}}{\mathbf{\mathcal{L}}}_{\boldsymbol{s},\boldsymbol{j}}\left({\boldsymbol{f}}_{\boldsymbol{\theta}}\right)\right)\end{array}} \end{equation*}

where $\boldsymbol{\alpha}$ denotes the learning rate of the inner loop, while ${\boldsymbol{\theta}}_{\boldsymbol{E}}$ and ${\boldsymbol{\theta}}_{\boldsymbol{p}}$ represent the parameters of the feature embedding network and the prediction network, respectively.

Next, the query set for cell line ${\boldsymbol{c}}_{\boldsymbol{j}}$ is input into the updated model ${\boldsymbol{f}}_{{\boldsymbol{\theta}}_{\boldsymbol{j}}^{\prime }}$, and the query set loss function ${\mathbf{\mathcal{L}}}_{\boldsymbol{q},\boldsymbol{j}}\left({\boldsymbol{f}}_{{\boldsymbol{\theta}}_{\boldsymbol{j}}^{\prime }}\right)$ is computed. In the outer-tier optimization, the total query loss across all ***N*** cell lines sampled in the episode, given by $\sum_{\boldsymbol{j}=\mathbf{1}}^{\boldsymbol{N}}{\mathbf{\mathcal{L}}}_{\boldsymbol{q},\boldsymbol{j}}\left({\boldsymbol{f}}_{{\boldsymbol{\theta}}_{\boldsymbol{j}}^{\prime }}\right)$, is calculated. Subsequently, the model’s initialization parameters $\boldsymbol{\theta}$ are updated via gradient descent as follows:


(10) \begin{equation*} {\displaystyle \begin{array}{c}\boldsymbol{\theta} \leftarrow \boldsymbol{\theta} -\boldsymbol{\beta} {\mathbf{\nabla}}_{\boldsymbol{\theta}}\sum_{j=1}^N{\mathbf{\mathcal{L}}}_{\boldsymbol{q},\boldsymbol{j}}\left({\boldsymbol{f}}_{{\boldsymbol{\theta}}_{\boldsymbol{j}}^{\prime }}\right)\end{array}} \end{equation*}

where $\boldsymbol{\beta}$ represents the learning rate for the outer-loop optimization. For each cell line ${\boldsymbol{c}}_{\boldsymbol{j}}$, the loss ${\mathbf{\mathcal{L}}}_{\boldsymbol{j}}\left(\boldsymbol{\theta} \right)$ is computed based on the downstream drug synergy prediction task, defined as the MSE between the true values $\boldsymbol{y}$ and the predicted values $\hat{\boldsymbol{y}}$:


(11) \begin{equation*} {\displaystyle \begin{array}{c}{\mathbf{\mathcal{L}}}_{\boldsymbol{j}}\left(\boldsymbol{\theta} \right)=\frac{\mathbf{1}}{\boldsymbol{m}}\sum_{\boldsymbol{i}=\mathbf{1}}^{\boldsymbol{m}}{\left({\boldsymbol{y}}_{\boldsymbol{i}}-{\hat{\boldsymbol{y}}}_{\boldsymbol{i}}\right)}^{\mathbf{2}}\end{array}} \end{equation*}

where $\boldsymbol{m}$ denotes the number of drug synergy samples used in the loss calculation.

##### Fine-tuning stage

In this stage, for drug synergy prediction tasks in new cell lines, we fine-tune the prediction network parameters using a small number of labeled samples to predict synergy scores for other drug pairs within these new cell lines. For each new cell line ${\boldsymbol{c}}_{\boldsymbol{n}\boldsymbol{ew}}\boldsymbol{\in}{\boldsymbol{C}}_{\boldsymbol{n}}$, the support set is defined as ${\boldsymbol{D}}_{\boldsymbol{s},\boldsymbol{new}}=\left\{\left({\boldsymbol{x}}_{\boldsymbol{s},\boldsymbol{new}}^{\left(\boldsymbol{i}\right)},{\boldsymbol{y}}_{\boldsymbol{s},\boldsymbol{new}}^{\left(\boldsymbol{i}\right)}\right)|\boldsymbol{i}=\mathbf{1},\boldsymbol{\cdots},\boldsymbol{K}\right\}$ and the query set as ${\boldsymbol{D}}_{\boldsymbol{q},\boldsymbol{new}}=\left\{\left({\boldsymbol{x}}_{\boldsymbol{q},\boldsymbol{new}}^{\left(\boldsymbol{i}\right)},{\boldsymbol{y}}_{\boldsymbol{q},\boldsymbol{new}}^{\left(\boldsymbol{i}\right)}\right)|\boldsymbol{i}=\boldsymbol{K}+\mathbf{1},\boldsymbol{\cdots},\boldsymbol{K}+\boldsymbol{Q}\right\}$. Leveraging the initialization parameters learned during the cross-tier optimization stage, which facilitate rapid adaptation to drug synergy prediction tasks across different cell lines, the feature embedding network ${\boldsymbol{\theta}}_{\boldsymbol{E}}$ extracts feature embeddings ${\boldsymbol{z}}_{\boldsymbol{s},\boldsymbol{new}}^{\left(\boldsymbol{i}\right)}$ and ${\boldsymbol{z}}_{\boldsymbol{q},\boldsymbol{new}}^{\left(\boldsymbol{i}\right)}$ from the support samples ${\boldsymbol{x}}_{\boldsymbol{s},\boldsymbol{new}}^{\left(\boldsymbol{i}\right)}=\left\{{\left({\boldsymbol{d}}_{\boldsymbol{A}},{\boldsymbol{d}}_{\boldsymbol{B}}\right)}^{\left(\boldsymbol{i}\right)},{\boldsymbol{c}}_{\boldsymbol{j}}\right\}\left(\boldsymbol{i}=\mathbf{1},\boldsymbol{\cdots},\boldsymbol{K}\right)$ and the query samples ${\boldsymbol{x}}_{\boldsymbol{q},\boldsymbol{new}}^{\left(\boldsymbol{i}\right)}=\left\{{\left({\boldsymbol{d}}_{\boldsymbol{A}},{\boldsymbol{d}}_{\boldsymbol{B}}\right)}^{\left(\boldsymbol{i}\right)},{\boldsymbol{c}}_{\boldsymbol{j}}\right\}\left(\boldsymbol{i}=\boldsymbol{K}+\mathbf{1},\boldsymbol{\cdots},\boldsymbol{K}+\boldsymbol{Q}\right)$, respectively. Subsequently, the prediction network ${\boldsymbol{\theta}}_{\boldsymbol{p}}$ estimates the drug synergy scores based on the support set embeddings ${\boldsymbol{z}}_{\boldsymbol{s},\boldsymbol{new}}^{\left(\boldsymbol{i}\right)}$ and computes the support set loss. The inner-tier optimization is performed according to Equation (9), yielding an updated prediction network ${\boldsymbol{\theta}}_{\boldsymbol{new}}^{\prime }$. Finally, the updated network ${\boldsymbol{\theta}}_{\boldsymbol{new}}^{\prime }$ predicts synergy scores ${\hat{\boldsymbol{y}}}_{\boldsymbol{new}}^{\left(\boldsymbol{i}\right)}$ for the query samples ${\boldsymbol{X}}_{\boldsymbol{q},\boldsymbol{new}}^{\left(\boldsymbol{i}\right)}$ in the new cell line, and the model performance is evaluated accordingly.

## Experiments and results

### Experimental settings and evaluation metrics

To evaluate the performance of MetaSynergy in the FSL setting, we randomly spilt the 106 cell lines into meta-training (85 lines, 80%) and meta-testing (21 lines, 20%) cell lines. Drug synergy samples from the meta-training cell lines were used to build the meta-training set, and those from the meta-testing cell lines were used for the meta-test set. Detailed information regarding the drug distribution across these sets is provided in Supplementary Fig. S1 and Table S1. This partitioning approach aligns with established benchmarking methodologies [28].

At the cross-domain pre-training stage, MetaSynergy was pretrained for 500 epochs using drug synergy samples from the meta-training set to learn the initial parameters of the feature embedding network. At the cross-tier meta optimization stage, we loaded the feature embedding network parameters obtained from the pre-training stage and randomly sampled 40,000 episodes from the meta-training set for training, with each episode consisting of 50 *K*-shot tasks (with *K* values of 5, 10, or 30). At the fine-tuning stage, we randomly sampled 40,000 *K*-shot tasks (with *K* = 5, 10 or 30) from the meta-testing set to evaluate the model's performance.

For the few-shot drug synergy prediction task, we compared the performance of MetaSynergy with four traditional machine learning methods (RF, XGBoost, SVM and KNN), the transfer learning method TFSynergy [19], previous drug synergy prediction methods for data-rich cell lines DeepSynergy [7] and MatchMarker [8], two optimization-based meta-learning algorithms (MAML [22] and BOIL [26]), and HyperSynergy [28]. Detailed implementation settings for all baseline methods are provided in Supplementary Appendix A.2. We assessed model performance on the regression task of predicting drug synergy scores using MSE, Spearman correlation coefficient (SCC), and coefficient of determination (R^2^). Classification performance for drug combination identification was evaluated using the area under the receiver operating characteristic curve (AUC) and the area under the precision-recall curve (AUPR). Definitions and mathematical formulations of these evaluation metrics are available in Supplementary Appendix A.3. To ensure fair comparison, all methods were evaluated using the same meta-training and meta-testing sets, repeated 20 times, following the evaluation protocol established by HyperSynergy [28].

### Results of MetaSynergy and other comparative methods in few-shot setting

**Figure 2:**
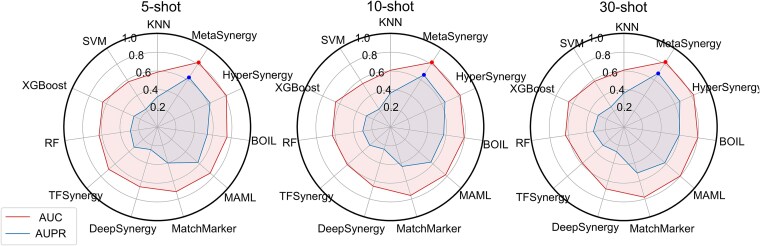
Results of MetaSynergy and other methods on the 5, 10, and 30-shot task in classification metrics.

To assess MetaSynergy’s performance in data-scarce cell lines, we conducted comparative evaluations against baseline methods on few-shot drug synergy prediction tasks (5-, 10-, and 30-shot scenarios). The experimental results are presented in Table 2 and Fig. 2. Additionally, Supplementary Fig. S3 presents the classification performance of MetaSynergy and other methods across different prediction thresholds.

**Table 2:** Results (mean ± STD) of MetaSynergy and other methods on few-shot setting in terms of three regression metrics.

Task	Metric	KNN	SVM	XGBoost	RF	TFSynergy	DeepSynergy	MatchMarker	MAML	BOIL	HyperSynergy	MetaSynergy
	MSE	0.181 ± 0.001	0.173 ± 0.005	0.148 ± 0.001	0.146 ± 0.003	0.168 ± 0.012	0.156 ± 0.006	0.145 ± 0.004	0.114 ± 0.002	0.110 ± 0.002	0.115 ± 0.002	**0.108 ± 0.001**
5-shot	SCC	0.333 ± 0.005	0.195 ± 0.009	0.294 ± 0.006	0.333 ± 0.002	0.290 ± 0.040	0.250 ± 0.010	0.356 ± 0.019	0.438 ± 0.003	0.443 ± 0.003	0.508 ± 0.004	**0.513 ± 0.011**
	${\mathrm{R}}^2$	−0.123 ± 0.003	−0.078 ± 0.031	0.109 ± 0.007	0.090 ± 0.002	−0.041 ± 0.073	0.029 ± 0.037	0.102 ± 0.025	0.191 ± 0.007	0.204 ± 0.010	0.181 ± 0.004	**0.225 ± 0.005**
												
	MSE	0.180 ± 0.001	0.163 ± 0.001	0.143 ± 0.002	0.146 ± 0.001	0.138 ± 0.012	0.158 ± 0.003	0.141 ± 0.001	0.111 ± 0.003	0.108 ± 0.002	0.111 ± 0.003	**0.107 ± 0.001**
10-shot	SCC	0.349 ± 0.005	0.220 ± 0.012	0.299 ± 0.008	0.336 ± 0.002	0.273 ± 0.010	0.237 ± 0.023	0.387 ± 0.015	0.445 ± 0.010	0.448 ± 0.002	0.530 ± 0.004	**0.530 ± 0.010**
	${\mathrm{R}}^2$	−0.121 ± 0.003	−0.013 ± 0.004	0.112 ± 0.013	0.094 ± 0.004	0.143 ± 0.054	0.018 ± 0.019	0.128 ± 0.006	0.209 ± 0.005	0.215 ± 0.003	0.210 ± 0.008	**0.235 ± 0.011**
												
	MSE	0.179 ± 0.001	0.153 ± 0.006	0.142 ± 0.001	0.143 ± 0.003	0.138 ± 0.007	0.154 ± 0.005	0.121 ± 0.005	0.110 ± 0.003	0.104 ± 0.002	0.105 ± 0.002	**0.104 ± 0.002**
30-shot	SCC	0.351 ± 0.001	0.253 ± 0.009	0.311 ± 0.006	0.349 ± 0.002	0.309 ± 0.011	0.269 ± 0.012	0.453 ± 0.014	0.478 ± 0.002	0.496 ± 0.003	0.545 ± 0.004	**0.552 ± 0.004**
	${\mathrm{R}}^2$	−0.110 ± 0.003	0.047 ± 0.038	0.118 ± 0.008	0.109 ± 0.005	0.145 ± 0.045	0.045 ± 0.030	0.251 ± 0.032	0.213 ± 0.004	0.249 ± 0.002	0.250 ± 0.007	**0.262 ± 0.005**

Note: Bold values indicate the best performance for each evaluation metric.

As shown in Table 2, MetaSynergy consistently outperforms all baseline methods across the 5-, 10-, and 30-shot regression tasks, achieving significant improvements: (i) 25.17–41.90% lower MSE, 51.86–163.08% higher SCC, and 54.96–122.03% higher R^2^ versus traditional machine learning methods; (ii) 22.46–35.71% MSE reduction, 76.90–94.14% SCC gain, and 64.34–80.69% R^2^ improvement versus transfer learning method TFSynergy; (iii) 14.05–25.52% lower MSE, 21.85–44.10% higher SCC, and 4.38–120.58% higher R^2^ versus data-rich synergy methods (DeepSynergy and MatchMarker); and (iv) 0.95–1.82% lower MSE, 0.98–17.12% higher SCC, and 4.80–24.31% higher R^2^ versus meta-learning methods (MAML, BOIL, and HyperSynergy). As shown in Fig. 2, MetaSynergy consistently surpasses all baselines across the 5-, 10-, and 30-shot classification tasks, achieving improvements of 0.9–7.7% in AUC and 3.7–14.6% in AUPR, even over the top-performing meta-learning methods (MAML, BOIL, and HyperSynergy). Furthermore, all models exhibited consistent performance gains with larger support sets (5 → 30-shot), confirming that increased sample size enhances task-specific learning and generalization.

These results demonstrate that: (i) pretraining on meta-training tasks followed by fine-tuning on limited meta-test samples is substantially more effective than directly mixing samples from both sets. (ii) Meta-learning methods surpass traditional approaches through task-agnostic and transferable initialization parameters, offering superior adaptability and sample size sensitivity. (iii) The inner-loop optimization of meta-learning relies heavily on the quality of the support samples, where selective fine-tuning prevents overfitting through restricted parameter updates, whereas larger support sets enable precise gradient estimation for robust cross-cell-line adaptation.

### Performance of MetaSynergy on lower cell line similarity few-shot setting

To further investigate the performance of MetaSynergy under biologically heterogeneous conditions in few-shot settings, as illustrated in Supplementary Fig. S2, we adopted the hierarchical clustering method by Zhang *et al.* [28] to partition 106 cell lines into meta-training (73) and meta-testing (33) groups with low inter-group similarity. Under this configuration, the similarity between meta-training and meta-testing cell lines ranged from 0.347 to 0.538, significantly lower than the similarity range observed with random partitioning (0.421–0.705).

**Figure 3:**
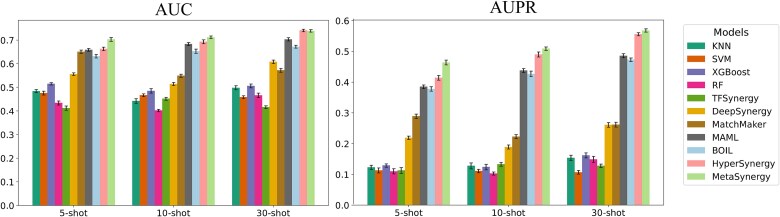
Comparison of AUC and AUPR performance between MetaSynergy and other methods on low-similarity few-shot setting.

Results in Fig. 3 show that all models exhibited performance declines under low-similarity conditions (reflecting increased task heterogeneity). MetaSynergy consistently surpasses all baselines in low-similarity scenarios (5/10/30-shot), achieving significant improvements of 2.74–6.03% in AUC and 2.16–12.08% in AUPR. These results underscore MetaSynergy’s superior generalization and adaptability to novel cell lines under significant distribution shifts, highlighting its stability in challenging few-shot scenarios.

### Results of MetaSynergy and other comparative methods in zero-shot setting

To evaluate MetaSynergy’s capability for drug synergy prediction in novel cancer cell lines under zero-shot learning conditions (where no labeled samples are available), we conducted comprehensive comparative experiments. To ensure a fair comparison, all models were trained exclusively on samples from the meta-training cell lines and directly evaluated on the meta-test cell lines without any fine-tuning. For meta-learning methods (MAML/BOIL/HyperSynergy/MetaSynergy), inner-loop optimization was disabled during both the meta-training and testing phases, effectively removing few-shot adaptation capabilities. Under these controlled conditions, MAML, BOIL, and MetaSynergy exhibited equivalent zero-shot performance; therefore, we report only MetaSynergy’s results.

As shown in Table 3, MetaSynergy significantly outperformed traditional machine learning methods, transfer learning (TFSynergy), and data-rich synergy predictors (DeepSynergy, MatchMarker). While demonstrating competitive performance against HyperSynergy (matching MSE with superior SCC/AUC), the narrowed advantage arises from the architectural designs. HyperSynergy’s static weight-generation architecture (prior-guided hypernetwork) enables effective zero-shot generalization. However, MetaSynergy’s dynamic optimization mechanism (inner-loop adaptation and fine-tuning) remains inactive under zero-shot constraints. This comparative analysis not only reveals fundamental methodological differences but also conclusively establishes the decisive contribution of the optimization mechanism to the performance of the proposed framework.

**Table 3:** Results (mean ± STD) of MetaSynergy and other methods on the zero-shot task in regression and classification metrics.

0-shot	MSE	SCC	${\mathrm{R}}^2$	AUC	AUPR
KNN	0.146 ± 0.001	0.242 ± 0.001	0.009 ± 0.001	0.602 ± 0.001	0.268 ± 0.001
SVM	0.235 ± 0.001	0.115 ± 0.001	−0.450 ± 0.006	0.596 ± 0.012	0.165 ± 0.001
XGBoost	0.159 ± 0.002	0.211 ± 0.005	0.011 ± 0.012	0.599 ± 0.008	0.252 ± 0.003
RF	0.192 ± 0.003	0.281 ± 0.003	−0.194 ± 0.016	0.605 ± 0.003	0.275 ± 0.001
TFSynergy	0.178 ± 0.001	0.282 ± 0.027	−0.103 ± 0.006	0.661 ± 0.001	0.330 ± 0.002
DeepSynergy	0.160 ± 0.005	0.238 ± 0.007	0.006 ± 0.030	0.654 ± 0.007	0.243 ± 0.005
MatchMarker	0.162 ± 0.016	0.224 ± 0.093	0.010 ± 0.010	0.696 ± 0.010	0.329 ± 0.005
HyperSynergy	0.108 ± 0.002	0.490 ± 0.004	**0.225 ± 0.005**	0.769 ± 0.003	**0.591 ± 0.004**
MetaSynergy	**0.108 ± 0.003**	**0.499 ± 0.003**	0.222 ± 0.003	**0.781 ± 0.001**	0.545 ± 0.002

Note: Bold values indicate the best performance for each evaluation metric.

### Performance of MetaSynergy on different meta-test cell lines

**Figure 4:**
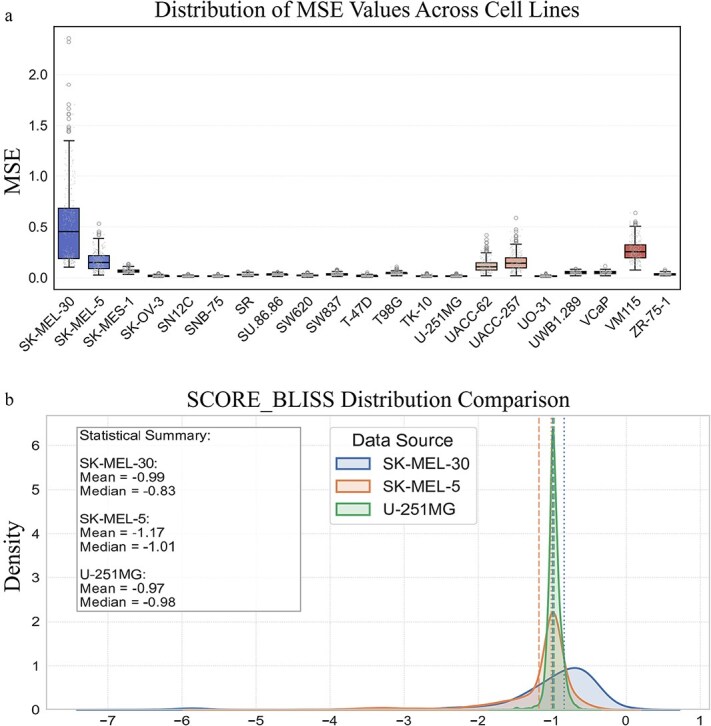
Results of MetaSynergy on different cell lines. (a) Box diagram of MSE of MetaSynergy on 21 metatest cell lines. (b) Distribution of synergy scores of all samples in SK-MEL-30, SK-MES-5, and U-251MG cell lines, respectively.

To systematically evaluate the predictive performance of MetaSynergy on different cell lines, we randomly sampled 300 test tasks from each meta-testing cell line to calculate the MSE for each task, and then evaluated the performance of MetaSynergy on different cell lines. Figure 4 presents the results of MetaSynergy on 21 meta-test cell lines. As shown in Fig. 4a, cell lines such as SK-MEL-30, SK-MEL-5, UACC-257, UACC-62, and WM115 exhibited relatively high MSE values, whereas others, such as U-251MG and TK-10 showed notably lower prediction errors.

To further investigate the performance differences, we visualized the distribution of synergy scores for the selected cell lines with contrasting MSE levels. Figure 4b shows the synergy score distributions for SK-MEL-30, SK-MEL-5, and U-251MG. We observed that the high-MSE cell line SK-MEL-30 showed a broader range of synergy scores, whereas the low-MSE cell line U-251MG exhibited more concentrated distributions. Moreover, by calculating the Pearson correlation between the average MSE of MetaSynergy on 300 few-shot tasks sampled from each cell line and the variation range of synergy scores within each cell line, we observed a strong positive correlation (r = 0.841, *P* < .001). These results suggest that greater variability in synergy scores increases the task difficulty, thereby reducing the predictive accuracy. The internal variability in synergy responses within a cell line appears to be a key factor influencing model performance, as higher dispersion likely introduces greater uncertainty and poses challenges for accurate prediction in biologically diverse contexts.

### Ablation studies of MetaSynergy

**Figure 5:**
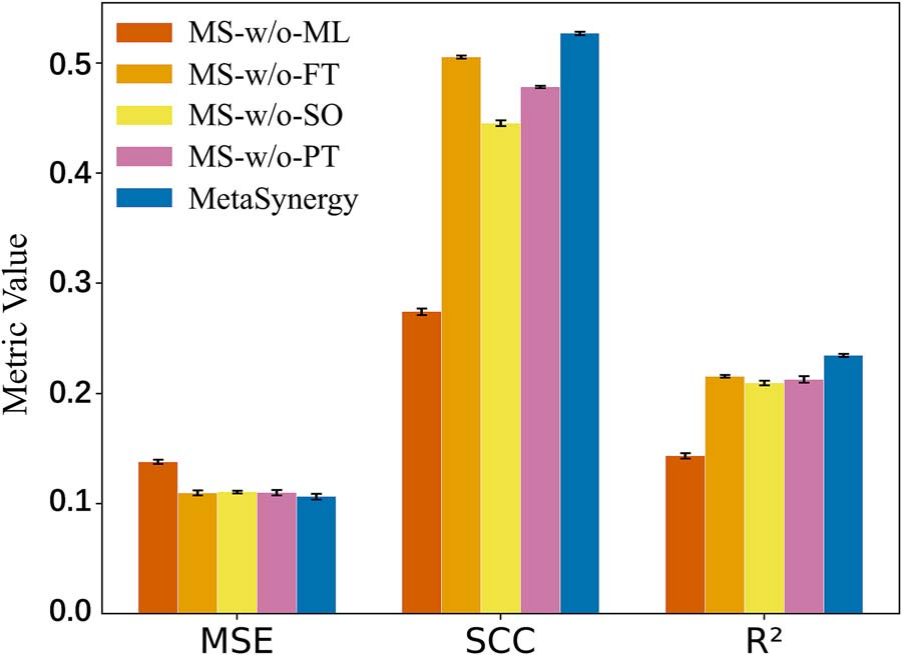
The ablation experimental results of MetaSynergy on 10-shot setting in terms of three regression metrics.

To evaluate the contribution of each core component in MetaSynergy, we conducted ablation studies on the 10-shot task, as shown in Fig. 5. Four architecturally distinct variants were assessed as follow:

- **MS-w/o-ML:** the R-CAMO meta-learning framework was removed, and the model was trained using standard supervised learning. During testing, fine-tuning was performed using ten labeled samples from each meta-test cell line.
- **MS-w/o-FT:** the fine-tuning stage was omitted, and the model was applied directly after cross-tier meta-optimization without adaptation to support samples.
- **MS-w/o-SO:** full parameter adaptation was enabled by updating both feature extraction network ${\boldsymbol{\theta}}_{\boldsymbol{E}}$ and prediction network ${\boldsymbol{\theta}}_{\boldsymbol{p}}$, instead of MetaSynergy’s selective strategy, which only fine-tunes the task-specific prediction network ${\boldsymbol{\theta}}_{\boldsymbol{p}}$.
- **MS-w/o-PT** eliminates the pretraining phase to isolate the effect of cross-domain knowledge transfer.

MetaSynergy consistently outperformed all ablated variants, underscoring the essential contributions of its core components. The removal of the meta-learning framework (MS-w/o-ML) resulted in a marked decline in generalization performance, emphasizing the significance of task-agnostic knowledge transfer. Omission of the fine-tuning stage (MS-w/o-FT) impaired task-specific adaptation, demonstrating its necessity for optimizing performance. Allowing full parameter updates (MS-w/o-SO) adversely affected generalization, confirming that selective adaptation of the prediction network mitigates overfitting. Excluding cross-domain pretraining (MS-w/o-PT) led to reduced accuracy, highlighting the importance of biologically informed model initialization. Overall, the R-CAMO framework effectively balances fine-tuning and generalization, preventing overfitting while preserving transferable knowledge, thereby enabling accurate few-shot drug synergy prediction.

### Case studies

We further employed MetaSynergy to predict novel synergistic drug combinations in three pharmacologically understudied cancer cell lines: A-673 (Ewing sarcoma), MFM-223 (breast cancer), and NCI-H1299 (non-small cell lung cancer). The model was trained on drug synergy data from 106 data-rich cell lines, and then used to predict synergy scores for drug pairs in these rare cellular contexts. To validate the predictions, we conducted a literature search in the PubMed database and identified that at least four predicted synergistic combinations per cell line have been supported by previously published studies (Table 4). For example, in A-673, the combination of Linsitinib, an IGF-1R and insulin receptor inhibitor, with Paclitaxel has shown improved outcomes in refractory ovarian cancer patients [32]. In MFM-223, Dovitinib enhances the cytotoxicity of Daunorubicin and Doxorubicin by disrupting the DNA damage checkpoint [33]. In NCI-H1299, Paclitaxel combined with Gefitinib inhibits growth in Gefitinib-resistant cells via apoptosis and senescence pathways [34] and MK-8776 increases Paclitaxel sensitivity by inhibiting drug efflux [35]. Additionally, Gefitinib and Cisplatin, combined with necitumumab and Gemcitabine, have demonstrated synergistic effects in lung squamous cell carcinoma by targeting EGFR signaling and DNA synthesis [36]. These results demonstrate MetaSynergy’s ability to generalize from data-rich to rare cancer cell lines, providing a valuable tool for prioritizing and accelerating the discovery of synergistic drug combinations in understudied malignancies.

**Table 4:** MetaSynergy-predicted and literature-validated drug synergy in understudied cell lines.

Cell Line	Drug A	Drug B	Synergy	PubMed ID
A-673	Linsitinib	Paclitaxel	0.1272	29454514
	SCH-900776	Paclitaxel	0.1032	31443367
	Oxcarbazepine	Paclitaxel	0.0574	18571399
	SB-225002	Paclitaxel	0.0330	30991054
MFM-223	Doxorubicin	Enzastaurin	0.1794	23866034
	Doxorubicin	Gsk-461364	0.1694	18583568
	Doxorubicin	Veliparib	0.1272	25066396
	Doxorubicin	Dovitinib	0.1189	24955955
NCI-H1299	Doxorubicin	Tigecycline	0.1705	34479918
	Paclitaxel	MK-8776	0.1374	31443367
	Paclitaxel	Gefitinib	0.0791	32831922
	Paclitaxel	Carboplatin	0.0656	16720915
	Gefitinib	Cisplatin	0.0351	30797492

## Conclusion

In this study, we presented MetaSynergy, a R-CAMO-based FSL framework for drug synergy prediction in data-scarce cellular contexts. The framework integrates structural representations from drug molecular graphs via GCNs and transcriptomic profiles via CNNs, which are fused into joint embeddings for predicting synergy. A stage-wise training strategy combining cross-domain pretraining, cross-tier meta-optimization, and task-specific fine-tuning leverages transferable meta-initialized features and hierarchical optimization to achieve both rapid adaptation and robust generalization. Our model outperformed most baseline methods in few-shot, zero-shot and low-similarity tasks, effectively addressing data scarcity challenges in drug synergy prediction through enhanced robustness and cross-cell-line generalizability. Moreover, ablation studies confirmed the pivotal role of the R-CAMO strategy in few-shot drug synergy prediction. Further analysis revealed a significant positive correlation between intercellular heterogeneity and model prediction errors. Notably, MetaSynergy successfully identified novel synergistic drug combinations in several understudied malignancies, such as Ewing sarcoma and breast cancer, highlighting its potential in precision oncology. This study opens two critical research avenues: (i) extending the framework to patient-derived samples for single-sample (1-shot) personalized synergy prediction, thereby bridging the gap between preclinical models and clinical applications, and (ii) incorporating interpretability modules to elucidate the biological mechanisms underlying predicted synergies. Together, these efforts will accelerate the clinical translation of precision oncology.

**Key Points**

- We propose MetaSynergy, a few-shot drug synergy prediction framework that integrates molecular graph of drugs and transcriptomic features of cell lines, to enable effective prediction in data-scarce cell lines through a Rapid Cross-tier Adaptation Meta-Optimization (R-CAMO)-based strategy.
- We introduce the cross-tier meta optimization mechanism, where the inner-tier refines task-specific parameters of the prediction network via gradient descent on target cell line data, while the outer-tier learns generalizable parameters transferable across cell lines, improving model adaptability.
- We designed a stage-wise training strategy to progressively transfer meta-knowledge from data-rich to data-scarce domains, allowing efficient fine-tuning with only a few labeled samples.
- MetaSynergy predicted novel synergistic drug combinations validated in several understudied malignancies, demonstrating its potential in drug synergy prediction for rare cancers and precision oncology treatment.

## Supplementary Material

Supplementary_Material_bbaf683

supp-Table_S1_bbaf683

## Data Availability

Code and data are openly available at the website of https://github.com/Emmnmusee/MetaSynergy.
