# Supplementary material for: Few-shot drug synergy prediction via rapid cross-tier adaptation meta-optimization
Source: Brief Bioinform. 2025 Dec 17;26(6):bbaf683. doi: 10.1093/bib/bbaf683 (PMC12710477; doi:10.1093/bib/bbaf683)
Supplement: Supplementary_Material_bbaf683 [file supplementary_material_bbaf683.docx]

Appendix A

**A.1 Datasets**

Drug synergy data in cancer cell lines were obtained from SYNERGxDB[1].SYNERGxDB integrates nine of the largest high-throughput anticancer drug combination screening studies, including NCI-ALMANAC, MERCK, MIT-MELANOMA, VIGAE, DECREASE, YALA-TNBC, YALA-PDAC, STANFORO, CLOUD. There are 477,839 drug combination-cell line data across 1,977 drugs and 151 cancer cell lines. The preprocessing protocol included three sequential quality controls: (i) excluding drugs without chemical structures (n=22), (ii) removing cell lines lacking transcriptomic profiles (n=26), and (iii) averaging experimental replicates. The final dataset comprised 427,710 drug combinations-cell line pairs acquired from 1,928 drugs and 106 cancer cell lines. Molecular topology information was obtained from PubChem as SDF files. Molecular was encoded as attributed graph, by the open-source cheminformatics library RDKit to convert the SMILES notation of the molecules into molecular graphs, where the nodes represent chemical atoms and the edges represent the chemical bonds between them. The gene expression profiles of the cancer cell lines were obtained from the Cancer Cell Line Encyclopedia (CCLE)[2] through Zhang, et al. [3], covering 17,135-dim protein-coding genes. Raw data processing was performed using microarray and RNA-seq platform pipelines: microarray data underwent multi-array normalization and log2-transformation, while RNA-seq data were converted from RPKM to TPM, followed by log2-transformation. Low-expressed genes (<1 TPM in >50% cell lines) were filtered, and batch correction was applied to ensure data consistency.

**A.2 Experimental settings**

For the few-shot drug synergy prediction task, we compared the performance of MetaSynergy with four traditional machine learning methods, transfer learning method TFSynergy [4], previous drug synergy prediction methods for data-rich cell lines DeepSynergy [5] and MatchMarker [6], two optimization-based meta-learning algorithms (MAML [7] and BOIL [8]), and HyperSynergy [3], which to our knowledge represents the first few-shot learning framework for drug synergy prediction using a prior-guided hypernetwork architecture. The details of the comparative methods are as follows:

- Traditional Machine Learning Methods: We evaluated KNN, SVM, XGBoost and RF. For these baselines, we used the sample embeddings generated by MetaSynergy as input features. The models were trained using drug synergy samples from the meta-training set along with *K* samples from each meta-test cell line, and subsequently used to predict the synergy scores of the remaining drug pairs in the same meta-test cell line.
- Transfer Learning Method: The core idea of transfer learning is to leverage knowledge from a source task to accelerate convergence on a target task, thereby reducing the requirement for large training datasets. In TFSynergy, the feature embedding network learns molecular representations from drug-related data including MACCS fingerprints, canonical SMILES strings, and target gene profiles, while simultaneously learning cell line representations based on cancer type and gene expression profiles. The prediction network then takes the embedded representations of drug pairs and cell lines as input to output synergy scores. During training stage, we first pretrained the feature embedding and prediction networks using drug synergy data from the meta-training set. Subsequently, the prediction network was fine-tuned with *K* samples from each meta-test cell line to predict synergy scores for the remaining drug pairs within the same cell line.
- Previous Drug Synergy Prediction Methods: DeepSynergy utilizes a feed-forward neural network that integrates drug chemical features (e.g., ECFP counts, physicochemical properties, toxicophore fingerprints) with cell line genomic profiles for regression-based prediction. MatchMaker adopts a dual-branch architecture where two parallel fully connected networks independently process the chemical and transcriptomic profiles of each drug in the pair, and their concatenated embeddings are passed to a third network to predict synergy scores. Both DeepSynergy and MatchMaker are adapted by training on a mixture of meta-training samples and *K* samples from each meta-test cell line.
- Optimization-Based Meta-Learning Algorithms: MAML and BOIL aim to learn a task-agnostic parameter initialization across tasks through bi-level optimization. In the inner loop, MAML updates all model parameters using *K* support samples to achieve rapid task adaptation, whereas BOIL updates only the parameters associated with sample-level feature representations, allowing task adaptation through refined embeddings. In the outer loop, both methods adjust the shared meta-initialization by evaluating post-adaptation performance across tasks. For few-shot drug synergy prediction, we adapted MAML to jointly update the feature encoder and prediction network based on the *K* samples, enabling effective adaptation to unseen cell lines. In contrast, BOIL was adapted to update only the feature encoder during the inner loop using the same *K* samples, thereby enhancing generalization through improved representation learning.
- HyperSynergy: The method pretrained the feature embedding network using drug synergy samples from the meta-training set. During the meta-learning phase, a variational inference network was employed to infer a task embedding for each meta-test cell line based on *K* samples. This task embedding was then passed to a hypernetwork, which generated the parameters of the prediction network. The resulting prediction network was used to estimate the synergy scores of the remaining drug combinations.

For fair comparison, all methods were evaluated using the same meta-training and meta-testing sets, repeating 20 times.

We performed a grid search of the hyperparameters of MetaSynergy by seeking both the minimum value of the loss function. There are four hyperparameters need to tune, including the hidden layer size of the graph neural network, the number of hidden layer neurons in the feature aggregation layer, the external and internal learning rates, as well as the Dropout value. Specifically, MetaSynergy turned the hidden layer size of the graph neural network from [78,128,156,256,312,512], the number of neurons in the feature aggregation layer's hidden layer from [128,156,256,312,512,1024,2048], and the external and internal learning rates from [0.1,0.01,0.001,0.0001]. Additionally, the dropout rate is selected from [0.0,0.2,0.3]. We ultimately selected [156, 312] as the hidden layer size for the graph neural network, [128, 256, 1024] as the number of neurons in the hidden layer of the feature aggregation layer, 0.001 as the internal learning rate, 0.01 as the external learning rate, and 0.2 as the Dropout value. All experiments are conducted on a Linux OS with 10GB NVIDIA RTX 2090 GPU, 40-core 2.4 GHz Intel Xeon CPU, and 128GB RAM. Our code is implemented based on PyTorch 2.1, CUDA 11.8, and PyTorch Geometric 2.4.

**A.3 Evaluation Metrics**

Given that our task involves a regression prediction problem, we employ a set of metrics to evaluate the model's performance. Specifically, these metrics include Mean Squared Error (MSE), Spearman Correlation Coefficient (SCC), and R-square (R²), each of which is calculated using Equations (1), (2), and (3), respectively. Specifically, MSE measures the discrepancy between observed and predicted drug synergy scores. SCC evaluates the correlation between those observed and predicted scores, while R² quantifies the model’s goodness-of-fit to a given dataset.

$$\begin{aligned} MSE=\frac{1}{n}\sum_{i=1}^{n} \left( \hat{y}_{i}-y_{i} \right)^{2}\#\left( 1 \right) \end{aligned}$$

$$\begin{aligned} SCC=1-\frac{6\sum_{i=1}^{n} d_{i}^{2}}{n\left( n^{2}-1 \right)}\#\left( 2 \right) \end{aligned}$$

$$\begin{aligned} R^{2}=1-\frac{\sum_{i=1}^{n} \left( \hat{y}_{i}-y_{i} \right)^{2}}{\sum_{i=1}^{n} \left( \bar{y}-y_{i} \right)^{2}}\#\left( 3 \right) \end{aligned}$$

Where $\boldsymbol{y}_{\boldsymbol{i}}$ represents the observed drug synergy score, ${\hat{\boldsymbol{y}}}_{\boldsymbol{i}}$ denotes the predicted score, and $\boldsymbol{n}$ is the total number of samples; $\bar{\boldsymbol{y}}$ is the mean of the observed drug synergy scores, and $\boldsymbol{d}_{\boldsymbol{i}}$ indicates the difference in ranking between the actually observed drug synergy score for the $\boldsymbol{i}$-th drug synergy sample and the predicted score by the model.

Furthermore, drug combinations with higher synergy scores are generally associated with better therapeutic efficacy and lower toxicity, making them promising candidates for clinical investigation. To evaluate MetaSynergy’s ability to identify such synergistic combinations, we binarized the continuous synergy scores by setting the top 10% as the positive class, following the threshold of -0.7569 as reported in [3][5]. Drug pairs with scores above this threshold were labeled as synergistic (positive class), while the remaining, including additive and antagonistic combinations, were labeled as non-synergistic (negative class).

Based on this binarization, we evaluated the classification performance using two metrics: **Area Under the ROC Curve (AUC)** and **Area Under the Precision-Recall Curve (AUPR)**. AUC quantifies the area under the Receiver Operating Characteristic (ROC) curve, which plots the True Positive Rate (TPR) against the False Positive Rate (FPR) at various threshold settings. AUPR measures the area under the Precision-Recall (PR) curve, which plots Precision versus Recall. The evaluation metrics were computed as follows:

$$\begin{aligned} True Positive Rate= \frac{TP}{TP+FN}\#\left( 4 \right) \end{aligned}$$

$$\begin{aligned} False Positive Rate=\frac{FP}{FP+TN}\#\left( 5 \right) \end{aligned}$$

$$\begin{aligned} Precision= \frac{TP}{TP+FP}\#\left( 6 \right) \end{aligned}$$

$$\begin{aligned} Recall= \frac{TP}{TP+FN}\#\left( 7 \right) \end{aligned}$$

where **TP** and **TN** refer to the number of correctly predicted synergistic and non-synergistic drug pairs, respectively, and **FP** and **FN** denote the number of incorrectly predicted synergistic and non-synergistic pairs, respectively.

**A.4 Problem formulation**

***Zero-shot learning task.*** Given a set of base cell lines $\mathbf{C}_{\mathbf{b}}$ and their drug synergy samples $\mathbf{D}_{\mathbf{b}}\mathbf{=}\left\{ \left( \mathbf{z}\mathbf{,y} \right) | \boldsymbol{z\in}\mathbf{Z}_{\mathbf{b}}\mathbf{,y}\mathbb{\in R} \right\}$ and a set of new cell lines $\mathbf{C}_{\mathbf{n}}$ that have no labeled drug synergy sample, the goal of zero-shot learning task is to obtain a function $\mathbf{f}_{\mathbf{nj}}\mathbf{:}\mathbf{Z}_{\mathbf{nj}}\mathbb{\to R}$ by learning a meta-function $\mathbf{F:}\mathbf{Z}_{\mathbf{j}}\boldsymbol{\to}\mathbf{f}_{\mathbf{j}}$ for predicting the drug synergy in new cell line $\mathbf{c}_{\mathbf{j}}\boldsymbol{\in}\mathbf{C}_{\mathbf{n}}$**.**

Appendix B


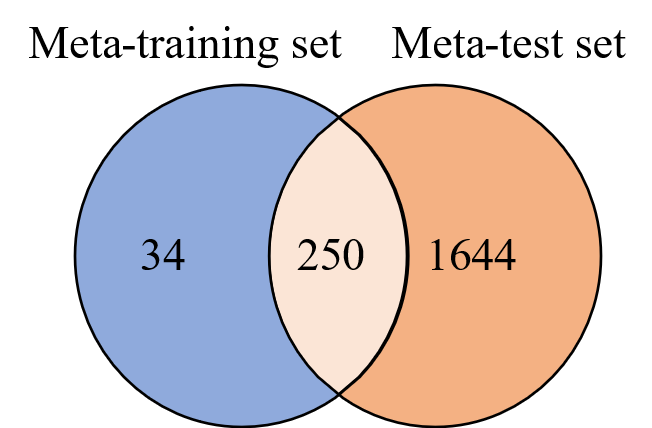


**Fig. S1** Venn diagram of drugs in meta-training set and meta-test set


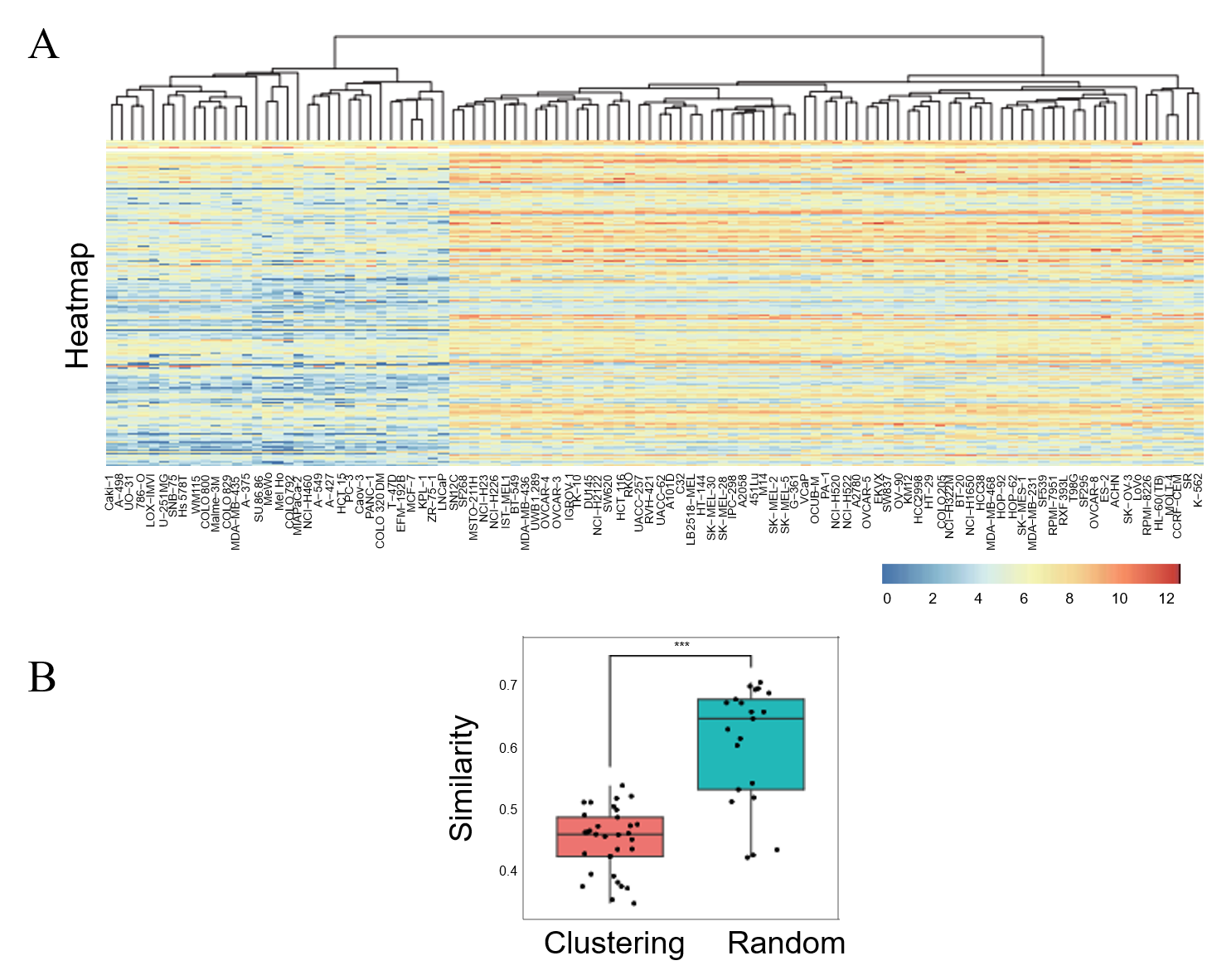


**Fig. S2** (A) Cluster Heatmap of the 106 cell lines. (B) Similarity between the meta-training cell lines and meta-test cell lines in the splits based on hierarchical clustering and random split.


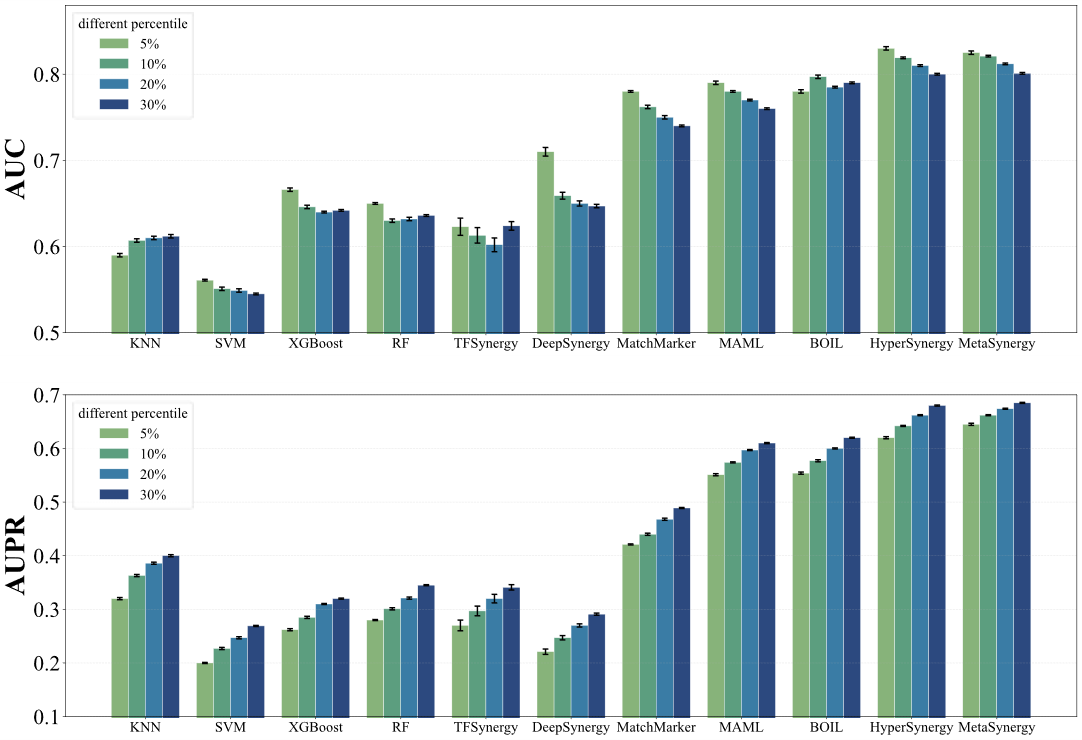


**Fig. S3** Results of MetaSynergy and other ten methods on 10-shot setting at different percentile thresholds in terms of AUC and AUPR.

[1] H. Seo *et al.*, "SYNERGxDB: an integrative pharmacogenomic portal to identify synergistic drug combinations for precision oncology," (in eng), *Nucleic Acids Res,* vol. 48, no. W1, pp. W494-w501, Jul 2 2020.

[2] M. Ghandi *et al.*, "Next-generation characterization of the Cancer Cell Line Encyclopedia," (in eng), *Nature,* vol. 569, no. 7757, pp. 503-508, May 2019.

[3] Q. Q. Zhang, S. W. Zhang, Y. H. Feng, and J. Y. Shi, "Few-Shot Drug Synergy Prediction With a Prior-Guided Hypernetwork Architecture," *IEEE Trans Pattern Anal Mach Intell,* vol. 45, no. 8, pp. 9709-9725, Aug 2023.

[4] Y. Kim, S. Zheng, J. Tang, W. Jim Zheng, Z. Li, and X. Jiang, "Anticancer drug synergy prediction in understudied tissues using transfer learning," (in eng), *J Am Med Inform Assoc,* vol. 28, no. 1, pp. 42-51, Jan 15 2021.

[5] K. Preuer, R. P. I. Lewis, S. Hochreiter, A. Bender, K. C. Bulusu, and G. Klambauer, "DeepSynergy: predicting anti-cancer drug synergy with Deep Learning," (in eng), *Bioinformatics,* vol. 34, no. 9, pp. 1538-1546, May 1 2018.

[6] H. I. Kuru, O. Tastan, and A. E. Cicek, "MatchMaker: A Deep Learning Framework for Drug Synergy Prediction," (in eng), *IEEE/ACM Trans Comput Biol Bioinform,* vol. 19, no. 4, pp. 2334-2344, Jul-Aug 2022.

[7] C. Finn, P. Abbeel, and S. Levine, "Model-Agnostic Meta-Learning for Fast Adaptation of Deep Networks," in *International Conference on Machine Learning*, 2017.

[8] J. Oh, H. Yoo, C. Kim, and S. Yun, "BOIL: Towards Representation Change for Few-shot Learning," in *International Conference on Learning Representations*, 2021.
